# Effects of short-term water velocity stimulation on the biochemical and transcriptional responses of grass carp (*Ctenopharyngodon idellus*)

**DOI:** 10.3389/fphys.2023.1248999

**Published:** 2023-08-31

**Authors:** Tingting Shu, Yan Chen, Kan Xiao, Hongtao Huang, Jingyi Jia, Zhaoxi Yu, Wei Jiang, Jing Yang

**Affiliations:** ^1^ Chinese Sturgeon Research Institute, China Three Gorges Corporation, Yichang, China; ^2^ Hubei Key Laboratory of Three Gorges Project for Conservation of Fishes, Yichang, China; ^3^ State Key Laboratory for Cellular Stress Biology, Innovation Centre for Cell Signaling Network, School of Life Sciences, Xiamen University, Xiamen, China; ^4^ Institute of Hydrobiology, Chinese Academy of Sciences, Wuhan, China

**Keywords:** grass carp, water velocity, ovary, hormones, transcriptome

## Abstract

Since 2011, ecological operation trials of the Three Gorges Reservoir (TGR) have been continuously conducted to improve the spawning quantity of the four major Chinese carp species below the Gezhouba Dam. In particular, exploring the effects of short-term water velocity stimulation on ovarian development in grass carp (*Ctenopharyngodon idellus*) is essential to understand the response of natural reproduction to ecological flows. We performed ovary histology analysis and biochemical assays among individuals with or without stimulation by running water. Although there were no obvious effects on the ovarian development characteristics of grass carp under short-term water velocity stimulation, estradiol, progesterone, follicle-stimulating hormone (FSH), and triiodothyronine (T3) concentrations were elevated. Then, we further explored the ovarian development of grass carp under short-term water velocity stimulation by RNA sequencing of ovarian tissues. In total, 221 and 741 genes were up- or downregulated under short-term water velocity stimulation, respectively, compared to the control group. The majority of differentially expressed genes (DEGs) were enriched in pathways including ABC transporters, cytokine-cytokine receptor interaction, ECM-receptor interaction, and steroid hormone biosynthesis. Important genes including *gpr4*, *vtg1*, *C-type lectin*, *hsd17b1*, *cyp19a1a*, *cyp17a1*, and *rdh12* that are involved in ovarian development were regulated. Our results provide new insights and reveal potential regulatory genes and pathways involved in the ovarian development of grass carp under short-term water velocity stimulation, which may be beneficial when devising further ecological regulation strategies.

## Introduction

Construction of hydraulic engineering not only significantly alters the natural hydrologic regime and water quality in the upstream and downstream of the dam (Poff and Schmidt, 2016; Chen et al., 2020), but also has undesirable ecological effects on aquatic species (Stone, 2016), and finally reduces the aquatic biodiversity (Liu et al., 2021). As the most severely affected biota at the top of the aquatic food chain, fish are often chosen as indicator species for determining the health status of the riverine ecosystems (Gao et al., 2009; She et al., 2023). Dam operations affect the spawning activity of native fish species and thus threaten aquatic populations and communities (Barbarossa et al., 2020; Mouchlianitis et al., 2021), and have been reported on rivers all over the world, such as the Madeira River, the Snake River, and the Colorado River in foreign basins (Finch et al., 2015; Cella-Ribeiro et al., 2017; McClure et al., 2020), as well as the Yangtze River, the Jinsha River, the Pearl River, the Han River, the Gan River, and the Lancang-Mekong River in domestic basins (Zhang C. et al., 2019; Zhang P. et al., 2019).

Fish reproduction is likely triggered by a variety of environmental factors, including flow velocity, water temperature, photoperiod, and dissolved oxygen (King et al., 2016; Buddendorf et al., 2017; Fellman et al., 2019). These environmental factors have a complex impact on fish spawning behaviors and gonadal development through adjusting the biological process of hormone synthesis and secretion. Among these environmental factors, water flow velocity is a key environmental factor that affects spawning and fertilization for fishes delivering drifting eggs (Lechner et al., 2014). Moreover, water flow velocity plays a crucial role in determining nutrient retention and oxygen delivery during fish spawning (McDonnell, 2000). Additionally, water flow velocity also affects gonadal development. Stimulated by water flows, fish generate impulses into the hypothalamus through sensory organs, and stimulate gonadotropin-releasing hormone (GnRH) release that direct acts on GnRH nerve terminals (Liu et al., 2021). Besides, the weakening of the water flow stimulus will lead to a decline in gonadal development among fish, resulting in a reduction of spawning quantity in watersheds, and potentially causing long-term cumulative differences in the population structure of fish stocks (Zhang W. et al., 2019).

The four major Chinese carp species, including grass carp (*Ctenopharyngodon idellus*), silver carp (*Hypophthalmichthys molitrix*), bighead carp (*Hypophthalmichthys nobilis*), and black carp (*Mylopharyngodon piceus*), play important roles in Chinese aquaculture and capture fisheries (Cao et al., 2015). A spawning site from Yichang to Chenglingji, situated at the middle reaches of the Yangtze River, is one of the most important natural reproduction zones of these Chinese carps, which accounting for 42.7% of the total spawning capacity along the river (Guo et al., 2011; Li et al., 2013a). However, in recent decades, due to the remarkable changes in hydrological conditions caused by the impoundment of the Three Gorges Reservoir (TGR), spawning behaviors and sexual maturation process of the four carp species have been severely hindered (Chen et al., 2021). In 2003, the number of fish eggs and larvae was only 10% of that in 2002, when the TGR began operation (Xie and Chen, 2001; Li et al., 2013b). Previous studies showed that water flow velocity suitable for grass carp spawning and sexual maturation mainly ranged from 0.33 to 1.50 m/s (Lin et al., 2022). Thus, providing essential ecological flows is widely recognized as an effective means of maintaining ecological integrity and restoring habitats for the spawning of major fish species in rivers (Stamou et al., 2018; Yang et al., 2019). However, the physiological mechanism of the response of natural reproduction to ecological flows is still unclear.

In this study, we used sexually mature female grass carp to conduct laboratory experiments to explore the effect of flow velocity on gonadal development in fish. The objective of the present study was to understand how flow velocity affect the gonadal development of grass carp. Specifically, we sought to a) explore the effect of short-term flow velocity on biochemical response, and b) analyze the possible regulatory mechanism of short-term flow velocity on the gonadal development of female grass carp. Our findings will provide a scientific basis for riverine ecosystem protection of the Yangtze River.

## Materials and methods

### Fish

Sexually mature females (n = 30, body length of 70.56 ± 3.13 cm, body weight of 5.16 ± 0.85 kg) and males (n = 30, body length of 68.40 ± 3.03 cm, body weight of 4.45 ± 0.70 kg) of grass carp were collected from Tengda Ecological Agriculture Development Co., Ltd. in Zhijiang City, Hubei Province, China. The grass carps were domesticated in Chinese Sturgeon Research Institute for 2 weeks, kept in a recirculating aquaculture system under controlled temperature conditions (21°C ± 0.5°C) with constant aeration, and fed in excess duckweed twice a day prior to the experimental trials.

### Experimental design

The experiment was performed in 20,000 L PVC circular tanks. Sixty grass carps were randomly divided into three groups with 10 females and 10 males, including the water velocity stimulation group, the hormone injection group, and the control group, labelled ZS, JS, and NS, respectively. Previous studies showed that suitable water flow velocity for grass carp spawning and sexual maturation mainly ranged from 0.33 to 1.50 m/s. However, even though ecological flows are provided, water mobility will weaken significantly in the middle and lower reaches of the Yangtze River. It was found that the flow velocity is rarely exceeding 0.5 m/s from April to June (Zhou et al., 2009; Wang et al., 2016; Liu et al., 2021). In some reservoir areas, the flow velocity cannot even reach 0.2 m/s (Xu et al., 2017). Therefore, we set a water velocity of 0.5 m/s in the ZS group. In the JS group, the females were injected with 2 mg/kg domperidone and 2.5 μg/kg LHRH-A2, at the base of the pectoral fin.

After 3 h, females were selected for sampling, including blood and ovaries. To avoid catching stress, fish were anesthetized by immersion with benzocaine (200 mg/L) (Geraylou et al., 2012; Wang et al., 2015), and then killed by a sharp blow to the head based on a previously described procedure (Hultmann et al., 2012). Blood samples were collected quickly from the caudal vein of each fish, then centrifuged at 3,000 g for 15 min at 4°C. The obtained serum samples were then transferred to −80°C for storage until enzyme linked immunosorbent assay (ELISA). Ovary tissues were also collected, and some of them were quickly fixed in 4% paraformaldehyde (PFA) in phosphate-buffered saline (PBS) at room temperature for 24 h for histological sections, and other ovaries were rapidly frozen in liquid nitrogen, then stored at −80°C until ELISA and RNA extraction.

### Histological analyses (hematoxylin and eosin (H&E) staining)

The fixed ovaries were dehydrated in graded ethanol solutions, and infiltrated with xylene. The sectioning and staining procedures were performed as described in a previous study (Lau et al., 2016). Briefly, the samples were embedded and processed for paraffin sectioning using a Leica RM2235 microtome (Leica Biosystems, Germany). Paraffin sections of 5 µm in thickness were mounted on slides, deparaffinized, rehydrated, and washed with ultrapure water. After staining with H&E, dehydrated, and mounted, the sections were observed and imaged by a Nikon Eclipse Ni-U microscope (Nikon, Japan). Scale bars are provided in the lower right corner of each image.

### Hormones measurement using ELISA

The concentrations of testosterone, estradiol, progesterone, and 17α,20β-dihydroxy-4-pregnen-3-one (DHP), as well as triiodothyronine (T3), thyroxine (T4), follicle-stimulating hormone (FSH), and luteinizing hormone (LH) in serum and ovaries were measured using commercial ELISA kits (mlbio, Shanghai). Briefly, 300 µL of serum was diluted to 500 µL with PBS, while 0.1 g ovary samples were isolated and homogenized in 1 mL PBS in a TGrinder H24R Tissue Homogenizer (TIANGEN, China). Following homogenization, the hormones were extracted with an organic solvent four times according to the manufacturer’s instructions. The layers were allowed to separate by vortex and centrifugation. Then the organic phase was transferred to a fresh tube and evaporated by heating to 30°C under a gentle stream of nitrogen. Finally, the extracts were dissolved in 200 µL ELISA buffer and the hormone concentrations were measured according to the manufacturer’s instructions.

### Transcriptome analyses

The ovary samples from grass carp were isolated and homogenized in a TGrinder H24R Tissue Homogenizer (TIANGEN, China), and total RNA was extracted by TRIzol reagent (Ambion, America) following the manufacturer’s instructions. RNA concentration was determined using NanoDrop One (Thermo Scientific, America), and the integrity and quality were assessed by RNA denaturing gel electrophoresis. High-quality RNA samples were selected for library construction. Using an Illumina NovaSeq 6000 system, RNA-Seq reads were generated by sequencing. Fastp (version 0.19.7) was used to generate clean reads. Then *de novo* assembly of clean reads was conducted with Trinity software (v2.6.6) and all assembled full-length sequences were named unigenes. All the unigenes were predicted and used for Blastx search and annotation against the NR, NT, KOG, SwissProt, PFAM, KEGG, and GO databases. After assembly, gene expression abundance was calculated using the Fragments Per Kilobase of transcript per Million mapped reads (FPKM) values. Differential expression analysis was performed using the DESeq2 package (v1.20.0) with a log2 fold change (FC) of 1.5 and a *p*-value cutoff of 0.05 (|log2FC|≥1.5, *p* < 0.05). GO function enrichment analysis and KEGG pathway enrichment analysis of differential gene sets were implemented using the Bioconductor R package clusterProfiler (v 3.18.1).

### Quantitative real-time PCR (qRT-PCR)

Independent RNA samples were extracted and used for cDNA synthesis for qRT-PCR to confirm the transcriptome results. RNA template with a content of 1.5 μg was used for reverse transcription to synthesize cDNA using the EasyScript^®^ One-Step gDNA Removal and cDNA Synthesis SuperMix kit (TransGen Biotech, Beijing) according to the manufacturer’s guidelines. All primers were designed using Primer-BLAST in the National Center for Biotechnology Information (NCBI), and the sequences are listed in Table 1. The primers for qRT-PCR were validated by agarose gel electrophoresis and DNA sequencing of PCR products. For amplification, the TransStart^®^ Tip Green qPCR SuperMix (TransGen Biotech, Beijing) and StepOnePlus™ real-time system (ABI, America) were used. All mRNA levels were calculated as the fold expression relative to the housekeeping gene, *β-actin*, *ef1a*, and *gapdh* and expressed as a fold change compared to the control group. Each sample was run in triplicate repeats and data analysis was performed using the ΔΔCt method (Schmittgen and Livak, 2008).

**TABLE 1:** Primer Sequences Used for qRT-PCR Analysis.

Gene	Direction^a^	Primer sequences (5′to 3′)	Primer length (bp^b^)	Amplicon length (bp)
*cyp17a1*	F	TGA​GGA​ACA​CAA​GGT​GAC​CTA​CAG	24	109
	R	GAC​ATC​ACG​AGT​GCT​GCT​G	19	
*hsd17b1*	F	GGCACCATCCGCACCA	16	111
	R	CTC​GTT​GAA​TGG​CAA​ACC​CT	20	
*slc12a2*	F	GTT​GCT​GAA​GAC​CTC​CGT​CA	20	208
	R	TAT​CAA​GTC​CCT​CTC​GCA​GT	20	
*vtg1*	F	GTG​ATG​CAC​CTG​CCC​AGA​TTG	21	159
	R	CCT​TGA​ACT​GAG​ACC​AGA​TAG​CCT​C	25	
*β-actin*	F	TGG​ACT​CTG​GTG​ATG​GTG​TGA​C	22	247
	R	GAG​GAA​GAA​GAG​GCA​GCG​GTT​C	22	
*ef1a*	F	AAA​ATT​GGC​GGT​ATT​GGA​AC	20	274
	R	TGA​TGA​CCT​GGG​CAG​TGA​A	19	
*gapdh*	F	CAC​CCA​TGG​CAA​GTA​CAA​GG	20	151
	R	GAC​ACC​GGT​AGA​CTC​CAC​AA	20	

^a^ F, forward; R, reverse.

^b^ base pairs.

### Statistical analysis

Statistical analysis was performed with GraphPad Prism 8.0 software (GraphPad software, America). All results were presented as mean ± standard deviation (SD) in each experimental group. Differences were determined using one-way ANOVA followed by Fisher’s least significant difference (LSD) test for multiple comparisons. For all statistical comparisons, *p* < 0.05 was considered statistically significant.

## Results

### Histopathology of ovary

**FIGURE 1:**
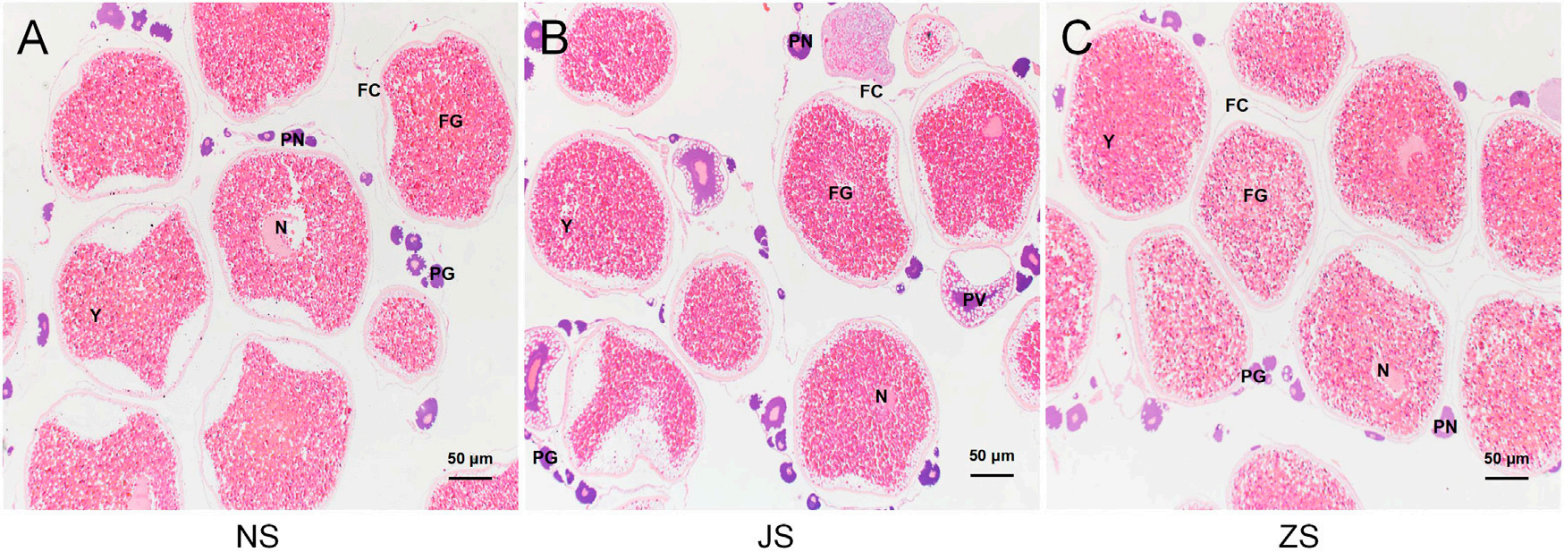
Histological sections of the ovarian status of grass carp. **(A)** NS group. **(B)** JS group. **(C)** ZS group. PG, primary growth follicle; PN, perinucleolar follicle; PV, previtellogenic follicle; FG, full-grown follicle; N, nucleus; Y, yolks; FC, follicle cells.

To determine the maturation level of grass carp gonads, we examined dissected ovaries by histological sections with H&E staining. Well-differentiated ovaries were observed in all groups, occupied by many full-grown follicles with normal reproductive characteristics (Figure 1). After short-term water velocity stimulation and the hormone injection, respectively, the development characteristics were similar to those of the control group. Specifically, the yolk filled the ovaries. Oocytes were easily identified by their large spherical nucleoli, each of which contained numerous nucleoli and a large cytoplasmic region bordered by a visible cell membrane. Each oocyte was a different size and was surrounded by a thin follicular cell layer. Most follicular cells contained ovoid nuclei and were stained with black, indicating that the fish used was in the pre-spawning period. No substantial effect was observed in the histological sections of the JS and ZS groups when compared to the NS group.

### Measurement of hormone concentrations

**FIGURE 2:**
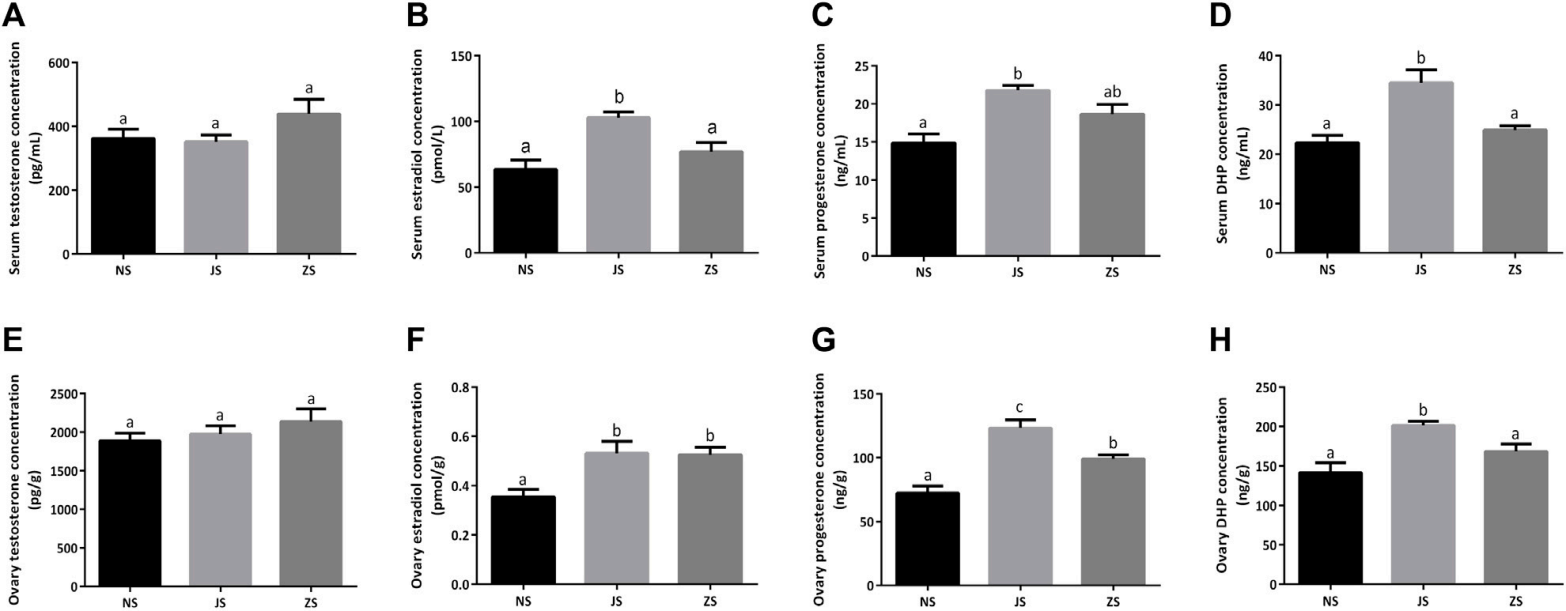
The sex steroid hormones measurements. Serum concentrations of testosterone **(A)**, estradiol **(B)**, progesterone **(C)**, and DHP **(D)** in the NS, JS, and ZS groups. Ovary concentrations of testosterone **(E)**, estradiol **(F)**, progesterone **(G)**, and DHP **(H)** in the NS, JS, and ZS groups. The letters in the bar charts represent significant differences.

The concentrations of several important sex steroids were measured in female grass carp. Serum and ovary testosterone concentrations did not differ among the three groups (Figures 2A,E). Serum and ovary estradiol, progesterone, as well as DHP concentrations were significantly higher in the JS group than those in the NS group (Figures 2B–D,F–H). However, only ovary estradiol and progesterone concentrations in the ZS group were significantly elevated (Figures 2F,G). Serum estradiol, progesterone, and DHP concentrations, as well as ovary DHP concentration in the ZS group were slightly increased, although there were not statistically significant (Figures 2B–D,H).

**FIGURE 3:**
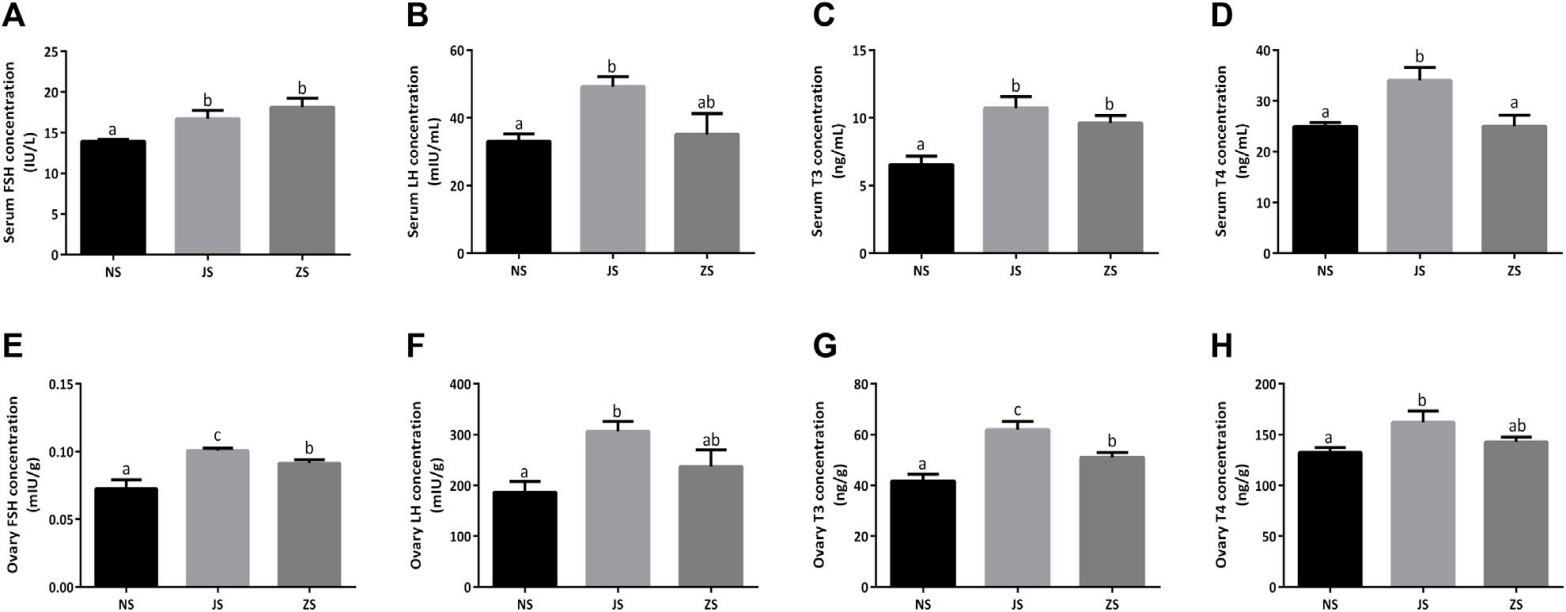
The gonadotropins and thyroid hormones measurements. Serum concentrations of FSH **(A)**, LH **(B)**, T3 **(C)**, and T4 **(D)** in the NS, JS, and ZS groups. Ovary concentrations of FSH **(E)**, LH **(F)**, T3 **(G)**, and T4 **(H)** in the NS, JS, and ZS groups. The letters in the bar charts represent significant differences.

Meanwhile, we examined serum and ovary gonadotropins, FSH and LH, in female grass carp. Both serum and ovary FSH and LH concentrations in the JS group were significantly increased compared to the NS group (Figures 3A,B,E,F). However, only enhanced serum and ovary FSH concentrations were observed in the ZS group (Figures 3A,E). LH concentrations were also compared, but did not show any significant differences. (Figures 3B,F).

Serum and ovary T3 and T4 concentrations were also evaluated in female grass carp. We observed that serum and ovary T3 concentrations in the JS and ZS groups were significantly higher than that in the NS group while elevated serum and ovary T4 concentrations were only observed in the JS group (Figures 3C,D,G,H).

### Overview of RNA-Seq

To identify the underlying molecular signaling pathways of short-term water velocity stimulation on the ovary transcriptional profile in grass carp, nine mRNA libraries were constructed and sequenced from NS, JS, and ZS ovary tissues using the Illumina NovaSeq 6000 system. All datasets from the Illumina sequencing platform are available in the NCBI Short Read Archive (SRA) database with the accession number (PRJNA977722). The main sequencing characteristics are listed in Table 2. A total length of 206,508,378 bp raw reads were obtained from the nine samples, and an average length of 21,593,012 bp clean reads were obtained from each sample after strict filtering. The Q20, Q30, and mapping rates for each group were within 96.90%–97.31%, 92.29%–93.18%, and 83.94%–85.97%, respectively, indicating that sequencing quality was acceptable for further analysis. Totally, 37,976 unigenes were acquired from *de novo* assembly. All unigenes were annotated by seven databases, including NR, NT, KOG, SwissProt, PFAM, KEGG, and GO databases (Table 3).

**TABLE 2:** Basic information of mRNA sequencing data of all samples in this study.

Sample	Raw reads		Clean reads	Q20 (%)	Q30 (%)	GC content (%)	Mapping ratio (%)
JS1	23,250,235		21,795,802	97.15	92.88	43.70	84.65
JS2	22,783,142		21,723,825	97.18	92.90	46.41	85.28
JS3	22,340,031		21,024,203	97.27	92.97	45.99	85.66
NS1	23,968,696		22,360,102	97.20	92.87	46.84	85.79
NS2	23,074,431		21,666,859	97.31	93.18	47.96	85.97
NS3	23,007,719		21,557,974	97.07	92.71	44.24	83.94
ZS1	23,045,475		21,720,377	97.04	92.62	45.22	84.70
ZS2	22,332,042		21,181,680	96.90	92.29	44.39	85.70
ZS3	22,706,607		21,306,290	97.19	92.91	45.47	84.48

**TABLE 3:** The statistics of annotation.

	Total unigenes	NR	NT	KOG	SwissProt	PFAM	GO	KEGG
Number of Unigenes	37,976	24,104	34,400	9,022	20,054	17,969	17,966	13,402
Percentage (%)	100	63.47	90.58	23.75	52.8	47.31	47.3	35.29

### Differentially expressed genes (DEGs) and pathway analysis

**FIGURE 4:**
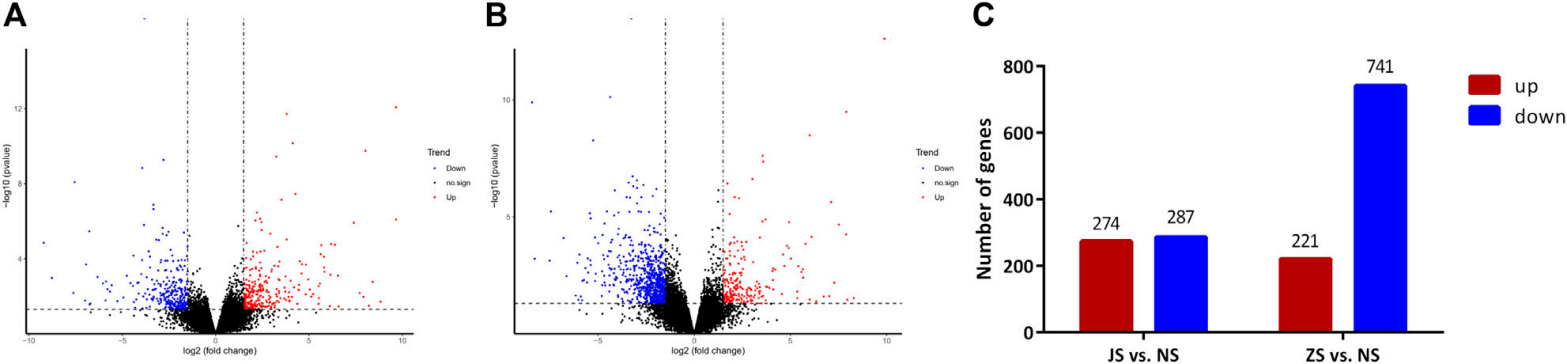
Volcano diagram of differential expression genes and numbers of DEGs in each comparison. **(A)** Volcano diagram of differential expression genes in JS vs. NS group. **(B)** Volcano diagram of differential expression genes in ZS vs. NS group. Significantly upregulated and downregulated genes are indicated in red and blue, respectively, and those not significantly different are in black. **(C)** The number of differentially expressed genes between different groups.

RNA sequencing analysis showed that the transcriptome profiles of JS and ZS groups were distinct from the NS group. Out of 21,248 annotated genes, we identified 561 differentially regulated genes between the JS and NS groups. Among the 561 regulated genes, 287 genes were highly expressed in the NS group while 274 genes were upregulated in the JS group (Figures 4A,C). Compared with expression levels in the NS group, 962 genes were differentially expressed in the ZS group, of which 221 and 741 were up- or downregulated in the ovary of treated fish (Figures 4B,C). Genes including *G protein-coupled receptor 4* (*gpr4*), *caspase a* (*caspa*), *solute carrier family 12*, *member 2* (*slc12a2*), *sterile alpha motif domain containing 9 like* (*samd9l*), *SRY-box transcription factor 4* (*sox4*), *forkhead box B1* (*foxb1*), *collagen type IV alpha 1 chain* (*col4a1*), *early growth response 1* (*egr1*), *vitellogenin 1* (*vtg1*), and *cytochrome P450 family 17*, *subfamily A member 1* (*cyp17a1*) were significantly regulated (Table 4). These results suggest that short-term water velocity stimulation had a significant effect on transcription in the ovary.

**TABLE 4:** Some significantly regulated genes.

Gene	Log2 fold change	
	JS vs. NS	ZS vs. NS
*gpr4*	8.02	7.91
*caspa*	5.83	5.41
*samd9l*	3.53	3.58
*slc12a2*	2.03	2.02
*sox4*	1.88	1.84
*foxb1*	−6.94	−4.4
*col4a1*	−4.56	−5.4
*egr1*	−2.91	−3.51
*vtg1*	−2.91	−2.87
*cyp17a1*	−2.11	−2.05

**FIGURE 5:**
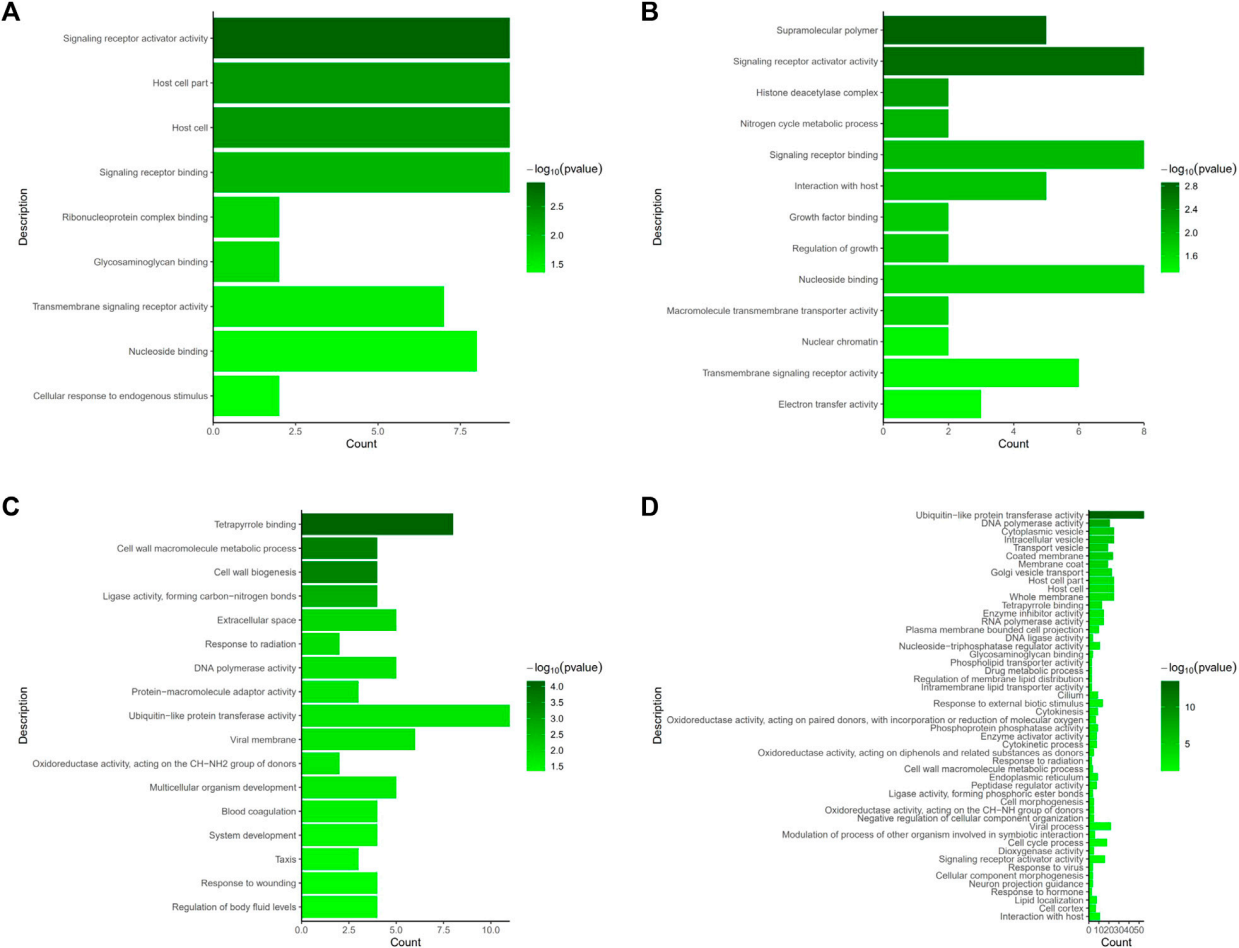
GO terms enrichment analysis of the differentially expressed genes. **(A)** Upregulated GO terms in JS vs. NS group. **(B)** Upregulated GO terms in ZS vs. NS group. **(C)** Downregulated GO terms in JS vs. NS group. **(D)** Downregulated GO terms in ZS vs. NS group.

We evaluated DEGs between the NS and JS groups, as well as the NS and ZS groups by GO and KEGG functional enrichment analyses. GO analysis revealed that genes from different signaling networks were significantly affected in the JS and ZS groups compared to the NS group. The important gene ontology upregulated in the JS and ZS groups included signaling receptor activator activity (*cxcl11.6*, *slc12a2*, *abcg5*), signaling receptor binding (*cxcl11.6*, *slc12a2*, *abcg5*), transmembrane signaling receptor activity (*gpr4*, *or131-2*, *tas1r1*, *cd84*), and nucleoside binding (*rab9a*, *rab1a*, *abcc2*) (Figures 5A,B, Supplemental Table S1, S2). Significantly downregulated gene ontology in the JS and ZS groups contained tetrapyrrole binding (*ba1*, *lama1*, *cyp17a1*, *cyp19a1a*), cell wall macromolecule metabolic process (*col4a1*, *pc*), DNA polymerase activity (*pol, znf180*), and ubiquitin-like protein transferase activity (*znf180*, *znf333*, *birc6*, *ercc6*, *rnf114*) (Figures 5C,D, Supplemental Table S1, S2).

**FIGURE 6:**
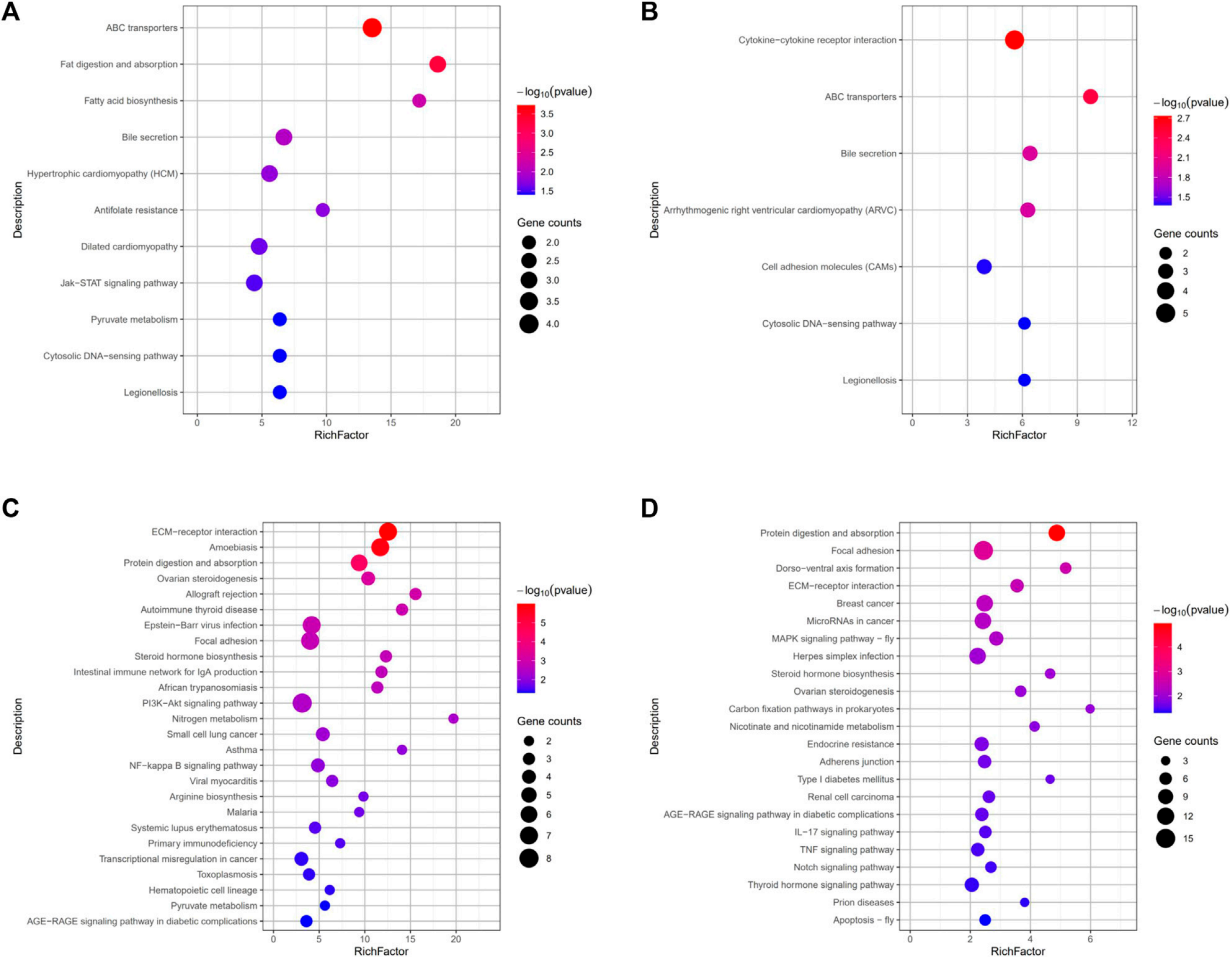
KEGG pathways enrichment analysis of the differentially expressed genes. **(A)** Upregulated pathways in JS vs. NS group. **(B)** Upregulated pathways in ZS vs. NS group. **(C)** Downregulated pathways in JS vs. NS group. **(D)** Downregulated pathways in ZS vs. NS group.

By comparing the DEGs to the KEGG pathway enrichment database, potential functions of the significant DEGs were analyzed to further understand the ovarian development of grass carp under short-term water velocity stimulation. For short-term water velocity stimulation treatment, 30 KEGG pathways (7 upregulated and 23 downregulated pathways) were significantly enriched in the ZS group (Figures 6B,D). Besides, compared with the NS group, 37 KEGG pathways (11 upregulated and 26 downregulated pathways) were significantly enriched in the JS group (Figures 6A,C). The KEGG enrichment analysis showed that genes involved in different pathways, such as ABC transporters (*abcc2*, *abcg5*), bile secretion (*abcc2*, *abcg5*), cytosolic DNA-sensing pathway (*caspa*, *cxcl11.6*), legionellosis (*caspa*, *rab1a*), ECM-receptor interaction (*lama1*, *col1a1*, *col4a1*, *col6a2*, *col1a2*, *col6a1*), protein digestion and absorption (*mme*, *col1a1*, *col4a1*, *col6a2*, *col1a2*, *col6a1*), ovarian steroidogenesis (*cyp17a1*, *cyp19a1a*, *hsd17b1*), focal adhesion (*lama1*, *col1a1*, *col4a1*, *col6a2*, *col1a2*, *col6a1*), steroid hormone biosynthesis (*cyp17a1*, *cyp19a1a*, *hsd17b1*), and AGE-RAGE signaling pathway in diabetic complications (*col1a1*, *col4a1*, *col1a2*) were differentially regulated in the JS and ZS groups (Figures 6A–D; Supplemental Table S3, S4).

### RNA sequencing data confirmation

**FIGURE 7:**
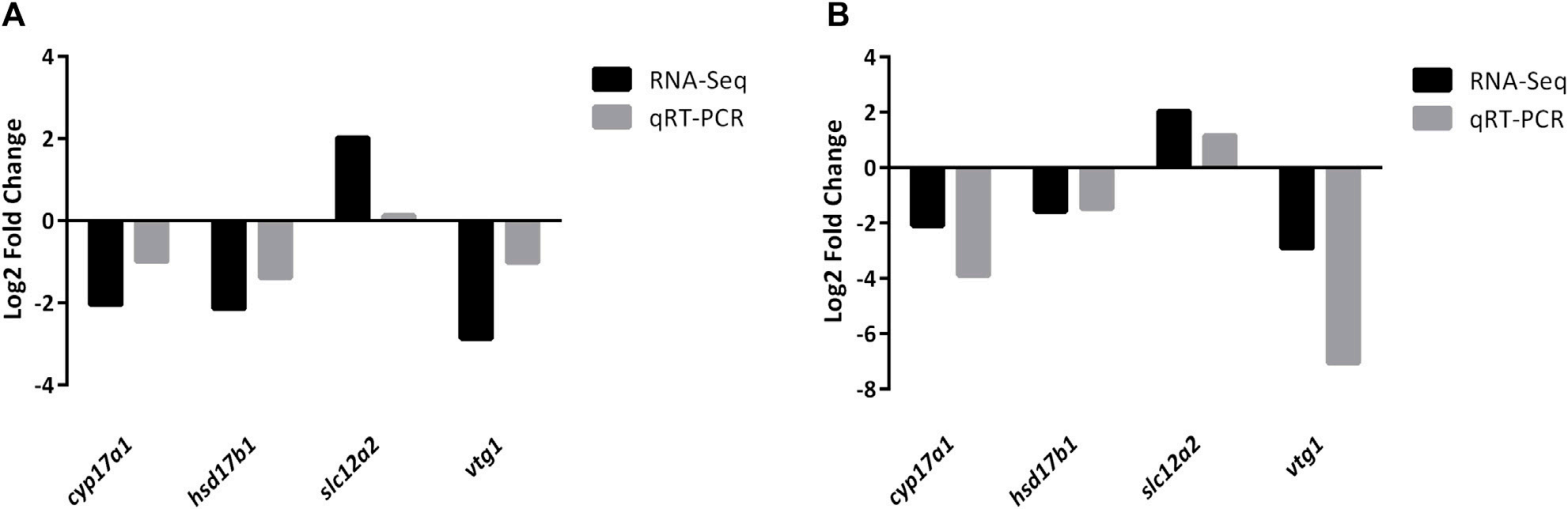
Validation of randomly selected four DEGs of RNA-Seq results using qRT-PCR analysis. All mRNA levels were calculated as the fold expression relative to the housekeeping gene *β-actin*. **(A)** Relative fold change of DEGs between qRT-PCR and RNA-Seq results in JS vs. NS group. **(B)** Relative fold change of DEGs between qRT-PCR and RNA-Seq results in ZS vs. NS group. Relative expression levels from the RNA-Seq results were calculated as log2FC values.

Four DEGs (*cyp17a1*, *hsd17b1*, *slc12a2*, and *vtg1*) were randomly selected to further validate the reliability of DEGs identified by RNA-Seq. The qRT-PCR results were consistent with those of RNA-Seq (Figure 7; Supplemental Figure S1, S2), indicating that the RNA-Seq data was accurate.

## Discussion

Because of the influence of large reservoirs distributed in the Yangtze River, the flow regime has been severely altered and flow velocity in the middle and lower river reaches is rarely exceeding 0.5 m/s from April to June (Zhou et al., 2009; Wang et al., 2016; Liu et al., 2021). In some reservoir areas, the flow velocity cannot even reach 0.2 m/s (Xu et al., 2017). To explore the effect of flow velocity on the ovarian development of female grass carp, we evaluated ovarian histology, hormone concentrations, and transcription levels of genes related to the ovaries. Our results revealed that even if ovarian development characteristics were not affected by short-term water velocity stimulation, the concentrations of sex steroids, gonadotropins, and thyroid hormones, as well as the transcriptional levels were significantly altered in female grass carp. These findings provide fundamental knowledge for technical support for ecological protection and restoration of hydraulic engineering.

Normal oocyte development and maturation are critical for successful reproduction in fish (Nakayama et al., 2004). However, no spawning activity was observed in the ZS group at a velocity of 0.5 m/s. Previous studies have shown that the required flow velocity for spawning differs greatly among different fish species. The determined triggering velocity of female silver carp was about 1.0 m/s in flume experiments (Chen et al., 2021), and female Atlantic salmon (*Salmo salar*)) constructs spawning redds in areas with an averaged flow velocity of 0.53 m/s (Beland et al., 1982). Generally, for the four major Chinese carp species, the velocity during the spawning period was 0.6–1.3 m/s, and the most appropriate velocity was 0.9–1.0 m/s (Dai et al., 2022).

The hypothalamus-pituitary-gonad (HPG) axis is responsible for fundamental regulation of all developmental stages of ovarian follicles, including progression to maturation or follicular atresia. And the pituitary gonadotropins, FSH and LH, which subsequently act on the ovary and regulate ovarian follicular development, maturity, steroidogenesis, and growth factor production, are the key players in the HPG axis (Patino et al., 2001; Nagahama and Yamashita, 2008; Zhang D. et al., 2022). In this study, we used serum and ovary testosterone, estradiol, progesterone, and DHP, as well as FSH, LH, T3 and T4 levels to represent gonadal development (Gadekar, 2014; Tucker et al., 2020), and measured these levels using ELISA. Normal reproductive functions in female fish are attributed to the sex steroid hormones, which mainly include testosterone, estradiol, progesterone, and DHP. By triggering germinal vesicle breakdown during final oocyte maturation, testosterone may contribute to oocyte growth and development (So et al., 1985). Moreover, testosterone is also involved in female steroidogenesis and acts as a substrate for aromatase during estradiol synthesis, which concentration is not stable in female fish (Barannikova et al., 2004). Estradiol is a crucial sex steroid hormone, and plays a significant role in stimulating the liver to produce the yolk precursor protein, vitellogenin, which is subsequently incorporated into the developing oocyte (Barannikova et al., 2004). Estradiol levels increase dramatically in the oocytes during vitellogenesis and decrease when vitellogenesis is complete (Amiri et al., 1996; Barannikova, 1999). Progesterone is a vital steroidogenic mediator for oocyte growth and maturation in female fish (Al-Hasawi, 2022). Our study showed that ovary estradiol and progesterone concentrations were all upregulated in the ZS and JS groups compared to the NS group (Figures 2F,G), indicating a positive effect of flow stimulation on fish gonad development. DHP acts as the most potent maturation-inducing steroid (MIS) in stimulating final oocyte maturation in fish (Amiri et al., 1999). In our study, serum and ovary DHP concentration in the ZS group was slightly increased (Figures 2D,H), although this was not statistically significant. Pituitary gonadotropins, FSH and LH, are major regulators of steroidogenesis by the ovary, resulting in the synthesis of sex steroid hormones that play critical roles in the orderly progression of growth and development of ovarian follicles (Harding et al., 2023). It is well known that thyroid hormones, T3 and T4, play a dominant role in oocyte development and final maturation and are well established in fish (Weber et al., 1992). We found that serum and ovary FSH and T3 concentrations in the ZS group were significantly elevated, as well as in the JS group (Figures 3A,C,E,G). However, no significant changes in LH and T4 levels were observed in the ZS group (Figures 3B,D,F,H). The underlying mechanism is unknown and requires further exploration. However, these data still show that short-term water velocity stimulation has an important influence on the ovarian development in female grass carp.

To understand the impact of short-term water velocity stimulation on gene expression of selected endocrine pathways and explore their underlying molecular mechanisms in fish, ovary samples from the NS, JS, and ZS groups were analyzed. Transcriptomic analysis of ovaries from the NS, JS, and ZS groups provided evidence that the water flow velocity is important for regulating genes from different signaling pathways. G protein-coupled receptors (GPRs), the largest membrane receptor family in eukaryotes, play a pivotal role in regulating various essential physiological and biochemical processes, including sexual maturation and reproduction (Flaherty et al., 2008; Nguyen et al., 2018). Ovarian development and maturation are controlled by many important factors, such as hormones and their receptors, which predominantly bind and activate GPRs on the cell surface, thereby initiating multiple downstream cascades (Zhang X. et al., 2022). We showed that *gpr4* was significantly upregulated in the ZS group, suggesting that it may regulate the ovarian development of grass carp under short-term water velocity stimulation. In fish oocytes, lectin may prevent polyspermy fertilization and participate in the formation of fertilization shell through binding with glycoproteins. Furthermore, lectin and vitellin are closely bound to ovomucin to form the basic structure of the vitellin outer membrane (Kido et al., 1992). In our study, the expression of *C-type lectin* was upregulated in the ZS group, suggesting that lectin affects ovarian development in grass carp under short-term water velocity stimulation.

Vitellogenin 1 (Vtg1) which plays a very important role in oocytes development was downregulated in the ZS group (Table 4). A large proportion of energy-related biomolecules from the liver, such as vitellogenin and lipids, are absorbed and utilized by the reproductive system (Della Torre et al., 2014; Zhang D. et al., 2022). In the turbot (*Scophthalmus maximus*), Xue et al. found that the ovary displayed a higher estradiol level and lower *vtg* expression, indicating that some other factors limit high *vtg* expression (Xue et al., 2018). Similar to the results of the present experiment, previous work in conger eel (*Conger myriaster*) also reported that flowing water could inhibit the gene expression of liver *vtg* and reduce VTG synthesis in the liver, which may promote lipid accumulation (Liu et al., 2022). In our study, flowing water stimulation may inhibit yolk accumulation during the ovarian development of grass carp. Retinol and its derivatives are known to play important roles in female reproductive processes, including follicular development, ovarian steroidogenesis, and oocyte maturation (Wang et al., 2022). Retinol dehydrogenase 12 (*rdh12*), a novel member of the microsomal short-chain dehydrogenase/reductase protein superfamily, has been identified as a key component in steroid metabolism (Keller and Adamski, 2007). In our results, *rdh12* expression was also decreased in the ZS group. Researchers have identified several genes that encode crucial enzymes in the steroidogenesis pathway, including *cyp19a1a*, *cyp17a1*, and *hsd17b1*, which could synthesize estradiol and progesterone, and play an important role in ovarian development and reproduction in fish (Fang et al., 2019; Li et al., 2021). Transcriptomic analysis showed that *cyp19a1a*, *cyp17a1*, and *hsd17b1* were also downregulated in the ZS group compared to the NS group. It has been proven that *cyp19a1a*, *cyp17a1*, and *hsd17b1* mRNAs showed a significant decrease when oocytes matured. Moreover, gene set enrichment analysis (GSEA) showed that the steroid hormone biosynthesis pathway was downregulated and that *cyp19a1a*, *cyp17a1*, and *hsd17b1* were core genes in this pathway (Dong et al., 2021). These findings revealed that these genes and this pathway play key roles in oocyte maturation. As previously described in ovoviviparous black rockfish (*Sebastes schlegeli*), the transcription level of *cyp19a1a* in ovary declined when the ovary developed from vitellogenic stage to ovulation stage during the reproductive cycle (Wen et al., 2014). A decreased *cyp19a1a* expression was also reported in amago salmon (*Oncorhynchus rhodurus*), rainbow trout (*Oncorhynchus mykiss*), and spotted scat (*Scatophagus argus*) during final maturation (Young et al., 1983; Gohin et al., 2011; Liu et al., 2015). In Japanese eel (*Anguilla japonica*), the expression of related transcripts *hsd17b1* and *cyp19a1a* declined at the migratory nucleus stage (Lai et al., 2022). These suggest that they can promote gonadal maturation in grass carp.

Utilizing KEGG pathway enrichment, the main biochemical metabolism and signal transduction pathways involved in genes can be identified. Generally, follicle development strongly depends on communication between germ cells and surrounding somatic cells through cytokine-cytokine receptor interaction, such as Kit and KitL (Matzuk et al., 2002; Saatcioglu et al., 2016). We observed a great number of genes in cytokine-cytokine receptor interaction obviously upregulated in ovaries of the ZS group (Figure 6B; Supplemental Table S4), indicating that the normal conservation between germ cells and somatic cells was already activated. Pathway analysis results indicated that 7 genes, including *lama1*, *col1a1*, *col1a2*, *col4a1*, *col6a1*, *col6a2*, and *col6a3*, enriched in ECM-receptor interaction were down regulated under short-term water velocity stimulation (Figure 6D; Supplemental Table S4). The pathways associated with ECM-receptor interaction play crucial roles in various biological processes, including cell migration, proliferation, follicle growth, and oocyte maturation (Berkholtz et al., 2006; Reing et al., 2009; Deng et al., 2022). Therefore, we speculate that these genes might have significant implications in the transition from the follicular development stage to the oocyte maturation stage. However, the specific mechanism remains unknown, and the functions of these genes in the reproductive cycle require further study.

## Conclusion

We investigated the ovarian development of grass carp under short-term water velocity stimulation by histology analysis, biochemical assays, and RNA-Seq technology. Although there was no obvious effect on the ovarian development characteristics of grass carp under short-term water velocity stimulation, estradiol, progesterone, FSH, and T3 concentrations were elevated. Totally, 962 DEGs with 741 downregulated genes and 221 upregulated genes were identified in transcriptome data. The key genes identified were enriched in ABC transporters, cytokine-cytokine receptor interaction, ECM-receptor interaction, and steroid hormone biosynthesis, which play an essential role in the response of the ovaries in grass carp to short-term water velocity stimulation. This study provides new insights into the ovarian development of grass carp under short-term water velocity stimulation. However, these transcriptomic data are still preliminary, and the function of the DEGs in reproductive cycle of fish species requires further investigation.

## Data Availability

The datasets presented in this study can be found in online repositories. The names of the repository/repositories and accession number(s) can be found below: https://www.ncbi.nlm.nih.gov/, PRJNA977722.
