# Supplementary figures and images for: Effects of short-term water velocity stimulation on the biochemical and transcriptional responses of grass carp (Ctenopharyngodon idellus)
Source: Front Physiol. 2023 Aug 31;14:1248999. doi: 10.3389/fphys.2023.1248999 (PMC10501314; doi:10.3389/fphys.2023.1248999)

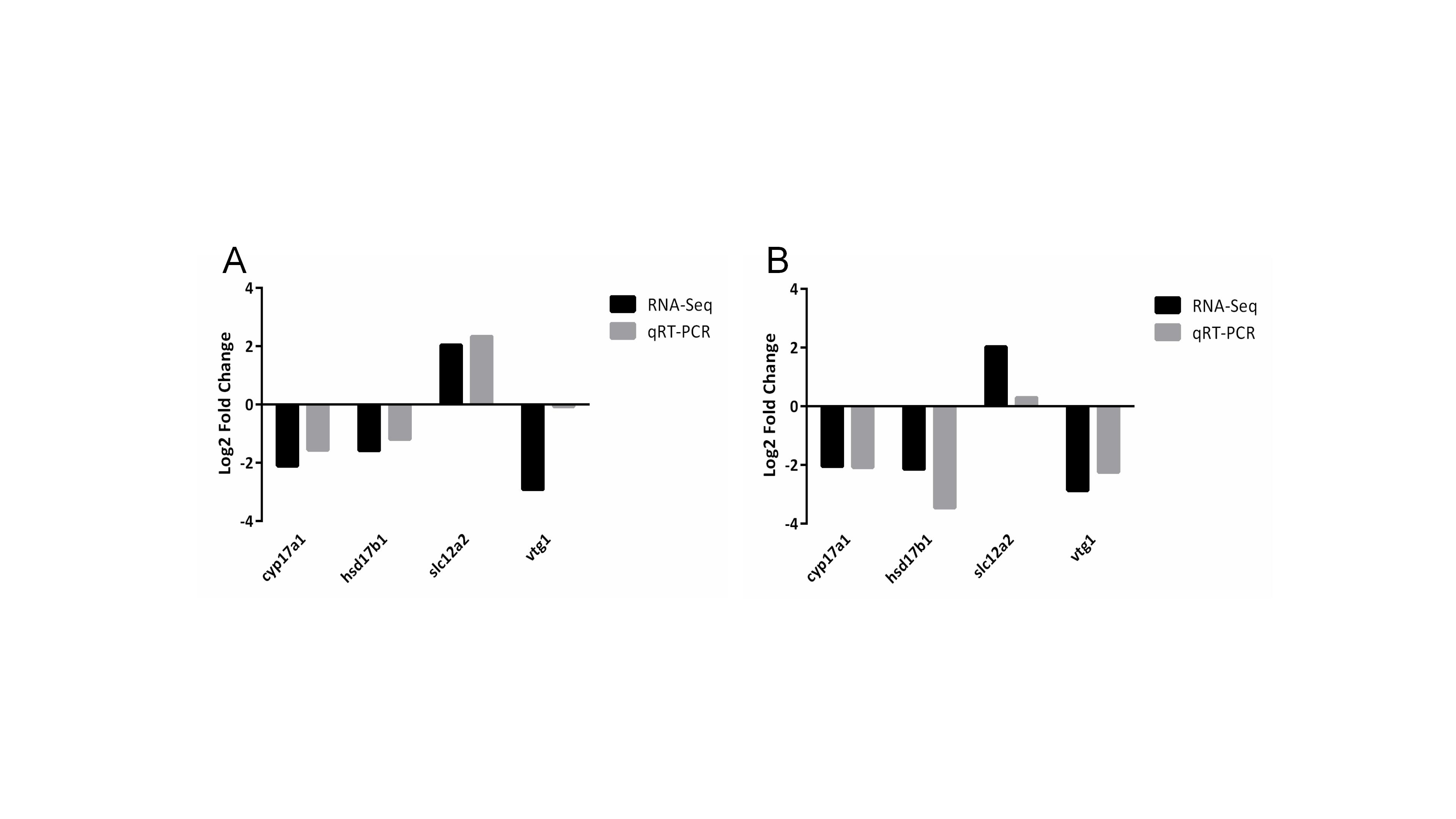

Supplement: Supplementary file 3 [file Image2.jpg]

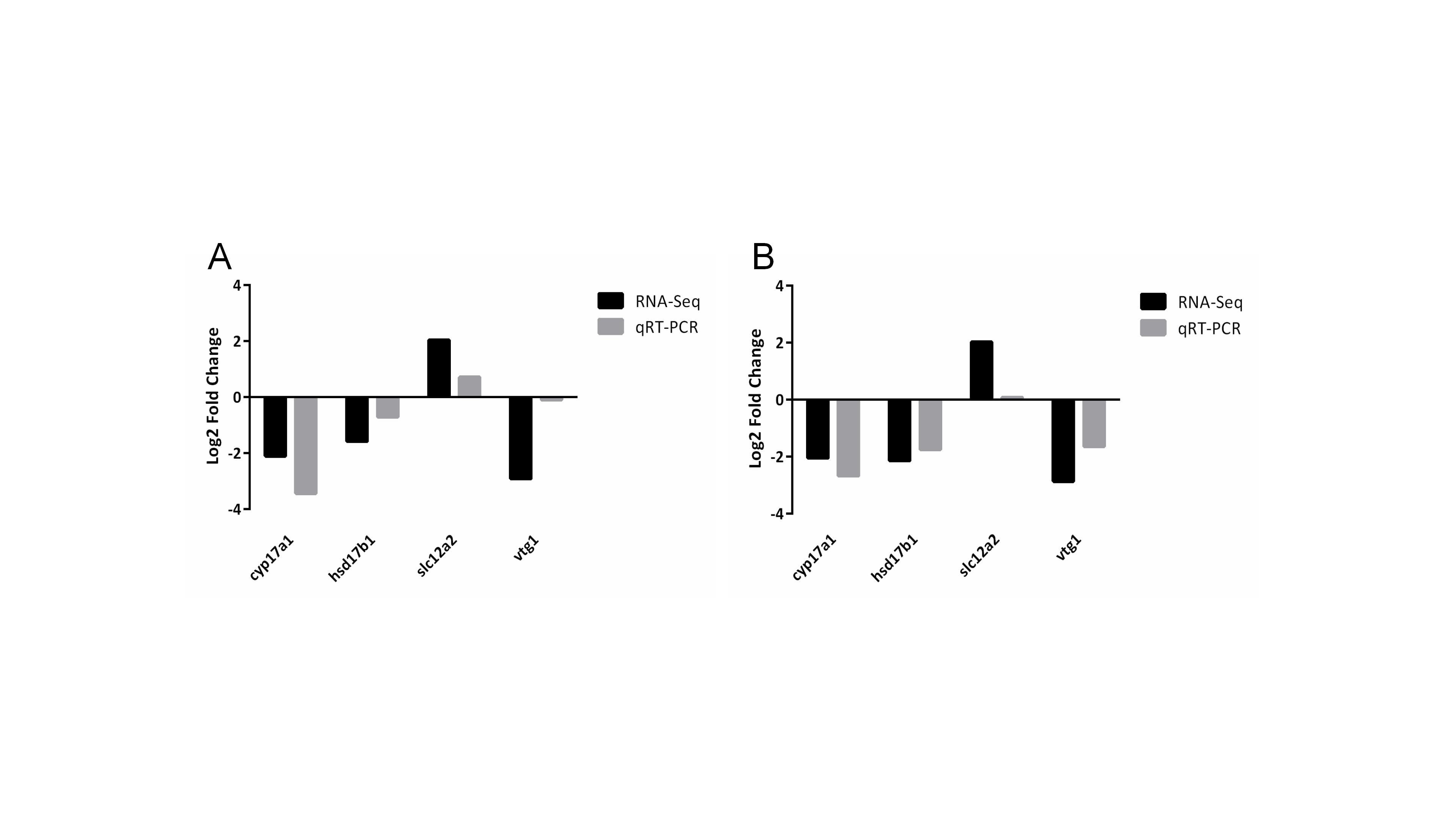

Supplement: Supplementary file 6 [file Image1.jpg]
